# Transmission dynamics model and the coronavirus disease 2019 epidemic: applications and challenges

**DOI:** 10.1515/mr-2021-0022

**Published:** 2022-02-28

**Authors:** Jinxing Guan, Yang Zhao, Yongyue Wei, Sipeng Shen, Dongfang You, Ruyang Zhang, Theis Lange, Feng Chen

**Affiliations:** Departments of Epidemiology and Biostatistics, School of Public Health, Nanjing Medical University, Nanjing, Jiangsu, China; China International Cooperation Center for Environment and Human Health, Center for Global Health, Nanjing Medical University, Nanjing, Jiangsu, China; Center of Biomedical BigData, Nanjing Medical University, Nanjing, Jiangsu, China; Department of Public Health, University of Copenhagen, Copenhagen, Denmark

**Keywords:** compartment model, coronavirus disease 2019, novel coronavirus pneumonia, SEIR, SIR, transmission dynamics model

## Abstract

Since late 2019, the beginning of coronavirus disease 2019 (COVID-19) pandemic, transmission dynamics models have achieved great development and were widely used in predicting and policy making. Here, we provided an introduction to the history of disease transmission, summarized transmission dynamics models into three main types: compartment extension, parameter extension and population-stratified extension models, highlight the key contribution of transmission dynamics models in COVID-19 pandemic: estimating epidemiological parameters, predicting the future trend, evaluating the effectiveness of control measures and exploring different possibilities/scenarios. Finally, we pointed out the limitations and challenges lie ahead of transmission dynamics models.

## Introduction

Infectious diseases are disorders caused by pathogenic organisms, including bacteria, virus, fungi or parasites. With appropriate mediums, they can spread between hosts (plants, animals and humans). The history of mankind is the one of combating infectious diseases. With the development of human civilization, towns and cities were built and connected. The tighter the connections between human individuals, the more possible that an infectious disease would spread.

It is critical to understand how the emerging infectious diseases transmit in a population, how the natural and social factors affect the transmission, and whether specific preventions and control measures will block the transmission path and can protect the susceptible population. Mathematical modeling has been a central part in the analysis of the transmission of infectious diseases for more than 100 years. As far as we know, the earliest mathematical model in infectious disease can be traced back to eighteenth century. In 1760, Daniel Bernoulli developed a model for smallpox to assess the effectiveness of vaccination in healthy people for preventing the smallpox virus. Later, he revealed the importance of smallpox vaccination, which extended the median life span by 14 years [1]. In 1906, to better understand the recurrence of measles epidemics, Hamer formulated a discrete time model, possibly the first model to propose that the incidence is related to the product of the densities of the susceptible and infected people in a population [2]. In 1911, Rose proposed to use differential equation models to study the transmission dynamic of malaria between mosquito and human [3]. His research illustrated that if the number of mosquitoes was reduced below some critical value, the epidemic of malaria would be controlled. This founding has made him win the Nobel Prize in Medicine for the second time. In 1927, Kermack and McKendrick proposed the first transmission dynamics model—SIR (susceptible, infectious and recovered) model, also called KM model, to study the bubonic plague in London from 1665 to 1666 and the plague in the island of Bombay from 1895 to 1906 [4]. Then, in 1932, they proposed SIR (susceptible, infectious and recovered) model and, based on previous works, obtained the epidemic threshold result that diagnosed whether an epidemic outbreak would occur.

This review will focus on the introduction of the transmission dynamics models, also called compartment models. We will firstly review the basic definitions of traditional SIR and SEIR (susceptible, exposed, infectious and recovered) models. Then, we will review how the SIR/SEIR model have been developed to account for the characteristics of severe acute respiratory syndrome coronavirus 2 (SARS-CoV-2), predict future trend and help decisions making.

## Transmission dynamics models

**Figure 1:**
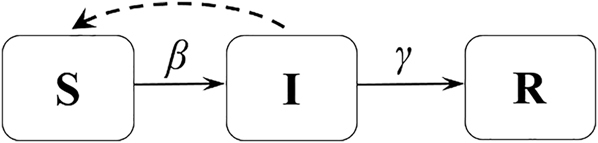
Diagram of a transmission dynamics model (SIR). S, susceptible; I, infected; R, recovered; *β*, transmission rate; 1/*γ*, the infectious period.

The transmission dynamic models of infectious diseases assume that the population can be partitioned into several non-overlap compartments. As shown by Figure 1, in a population, each individual is in one of the three compartments, Susceptible (*S*), Infected (*I*) and Recovered (*R*). At the very beginning of an epidemic, some (maybe only a few) individuals are infected by some pathogen while others are not infected but susceptible to the disease. The susceptible individuals have some probability to be infected after contacting the pathogen through some medium (as indicated by the dashed line in Figure 1), while some infected individuals may recover after a few days and could not pass the pathogen to other susceptible individuals.

**Figure 2:**
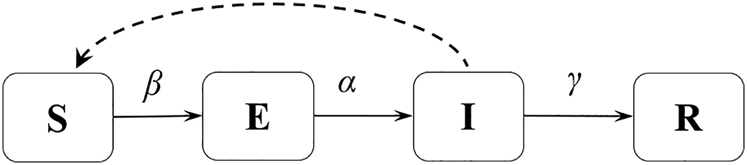
Diagram of SEIR model. S, susceptible; E, exposed; I, infected; R, recovered; *β*, transmission rate; 1/*α*, the incubation period; 1/*γ*, the infectious period.

An extension to SIR model is the SEIR model. As shown by Figure 2, a status of exposed (*E*) individuals in the incubation period is inserted between S and I to reflect that it may need some time for an infected individual to build sufficient ability for onward transmission.

This simple compartmentalization makes it possible to use a set of simple ordinary differential equations to capture the dynamic transitions among different infectious status. For a closed system, the ordinary differential equations for the SEIR model are as follows:

$$\left\{ \begin{array}{l} \frac{dS}{dt}=-\frac{\beta SI}{N}\\ \frac{dE}{dt}=\frac{\beta SI}{N}-\alpha E\\ \frac{dI}{dt}=\alpha E-\gamma I\\ \frac{dR}{dt}=\gamma I\\ N=S+E+I+R \end{array}\right.$$

where *S*, *E*, *I* and *R* are the numbers in these compartments. The three per-capital rates included in the transmission model (*α*, *β* and *γ*) determine the transmission progress between different diseases status, and can be easily used to generate important parameters that describe the characteristics of an epidemic. As an example, a crucial parameter in transmission dynamics models is the basic reproductive number (*R*_0_), which is the number of next generation cases infected by one infected individual on average when this infected individual comes into a population where all individuals are susceptible [1]. If *R*_0_>1, the transmission in the fully susceptible population would be started. If *R*_0_<1, the infectious disease will not spread. *R*_0_ of SEIR model is calculated by

$$R_{0}=\frac{\beta }{\gamma }$$

Since 1950s, the theories of transmission dynamics models have been greatly developed. Transmission dynamics models were extended to involve aspects such as the incubation period of the disease (with or without infectiousness), quarantine, stage of infection, immunity (including loss of immunity) of susceptible and infected individuals, age structure, birth, natural death, population migration, immunity of prognostic individuals, vertical transmission, spatial spread, disease vectors and other conditions and have been used in a variety of human diseases such as measles, chickenpox, smallpox and so on [1].

## Transmission dynamics models in the coronavirus disease 2019 pandemic

Since December, 2019, a cluster of pneumonia cases infected by SARS-CoV-2 was reported in Wuhan and soon became a Public Health Emergency of International Concern [5]. Up to December 12, 2021, coronavirus disease 2019 (COVID-19) has resulted in over 269 million confirmed cases and 5.3 million deaths.

Traditional SEIR model had been applied in the modelling of the COVID-19 pandemic. However, the characteristics of SARS-COV-2 itself, as well as the interventional policies adapted by governments, limited the use of SEIR model. For COVID-19, previous studies had developed a series of models with different extensions which could be categorized into three categories: compartment extension, parameter extension and population-stratified model (Table 1).

**Table 1:** Extensions of transmission dynamics model.

Type of extension	Extending
Compartment extension	Asymptomatic
	Presymptomatic
	Unascertained
	Disease severity stage
	Hospitalized
	Quarantine
Parameter extension	Effect indicator for control measures
	Time-varying transmission rate
	Relative transmission rate of E/A vs. I
	Population flow
Population-stratified model	Geographical unit
	Demographic information

E, exposed; A, asymptomatic; I, infected.

With the inclusion of additional compartments, it is possible to account for additional disease stage (presymptomatic, asymptomatic, ascertained and unascertained) and status (mild, moderate, severe and critically ill). It is also possible to incorporation the information of interventional policies, such as wearing masks, universal screening, quarantine and hospitalization, etc.

### Extension of SEIR models with compartments for additional disease status

**Figure 3:**
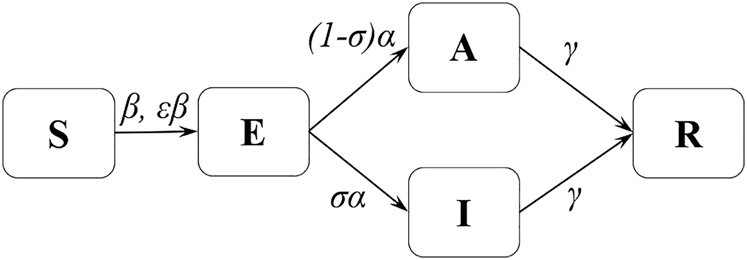
Diagram of SEAIR model. S, susceptible; E, exposed; A, asymptomatic; I, infected; R, recovered; *β*, transmission rate; *ε*, the relative transmission factor compared with symptomatic cases; 1/*α*, the incubation period; σ, the proportion of symptomatic cases; 1/γ, the infectious period.

**Figure 4:**
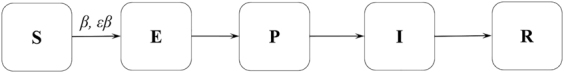
Diagram of SEPIR model. S, susceptible; E, exposed; P, presymptomatic; I, infected; R, recovered; *β*, transmission rate; *ε*, the relative transmission factor compared with symptomatic cases.

As an example, previous studies had revealed the infectivity of asymptomatic and presymptomatic cases [6], [7], [8], [9]. Some studies developed modified SEIR/SIR model with an additional compartment representing asymptomatic cases (*A*), named SEIAR model (Figure 3), or presymptomatic cases (*P*), named SEPIR model (Figure 4) [10], [11], [12].

In the SEPIR model, infected individuals in incubation period are further divided into two compartments: exposed compartment (*E*), exposed but without infectiousness, and presymptomatic compartment (*P*) (Figure 4) [12]. For the two additional compartments of infection, *A* and *P*, they have different transmission rates compared with those infection with symptom (*I*): the transmission rate at which a susceptible individual becomes infected due to contact with symptomatic infections, *β*; and the transmission rate due to infected individuals who were infected without symptom or in the incubation period, *εβ*, *ε* is the relative transmission factor compared with symptomatic cases.

**Figure 5:**
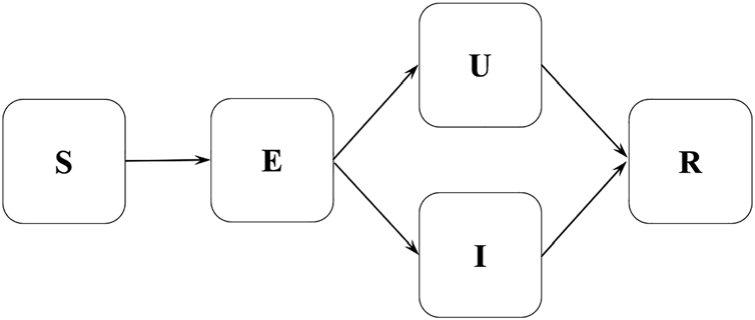
Diagram of SEIRU model. S, susceptible; E, exposed; I, infected; R, recovered; U, unascerntained.

In the early epidemic of COVID-19 in Wuhan, some infected cases were indeed unascertained due to lack of symptoms and the limited screening capacities. Similar to the SEIAR model, an additional compartment of unascertained cases (*U*) was then inserted into the SEIR model, leading to the SEIRU model [13]. This model was successfully applied in the exploration on how many cases were unascertained due to insufficient nucleic testing capacity (Figure 5).

**Figure 6:**
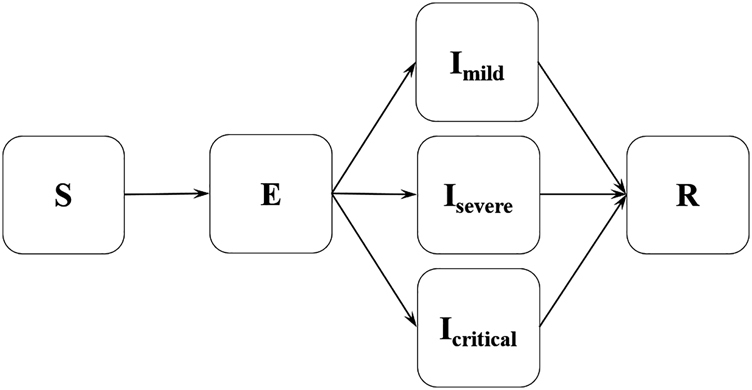
Diagram of SEIR model with severity compartments. S, susceptible; E, exposed; _I_mild, infected individuals with mild symptom; _I_severe, infected individuals with severe symptom; _I_critical, infected individuals with critical symptom; R, recovered.

As patients at different disease stage may have different ability to forward the transmission, another extension of SEIR model divided the *I* compartment into three compartments denoting different disease severities: infected individuals with mild symptom (*I*_*mild*_), infected individuals with severe symptom (*I*_*severe*_) and infected individuals with critical symptom (*I*_*critical*_) (Figure 6) [14].

### Extension of SEIR models with compartments for interventions

**Figure 7:**
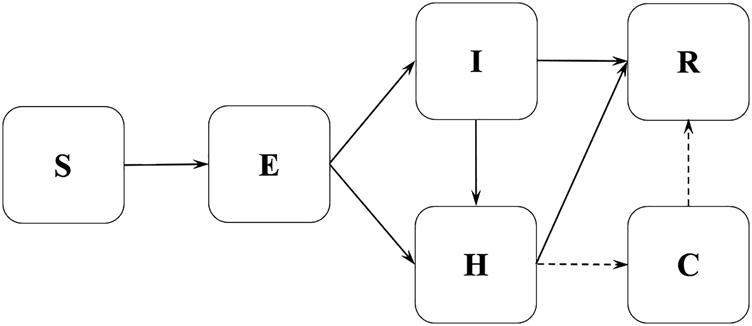
Diagram of SEIRHC model. S, susceptible; E, exposed; I, infected; R, recovered; H, in hospital; C, in ICU.

In addition to different disease status and stages, interventions on the individuals would also change the transmission dynamics of the infectious diseases. As a result, a series of models incorporating the information on hospitalization, even the admission to the intensive care unit (ICU) had been developed [15, 16]. In these models, some of the exposed individuals with symptoms would move to the *H* compartment to obtain health cares. Some of the critically ill patients would even move to the ICU. While others with no/mild symptom would enter *I* compartment and become recovery naturally (Figure 7) [16].

**Figure 8:**
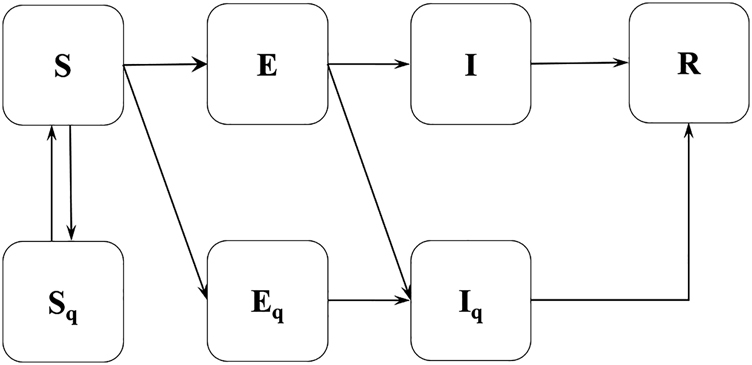
Diagram of SEIR^+^Q model. S, susceptible; E, exposed; I, infected; R, recovered; S_q_, quarantined susceptible; E_q_, quarantined latent cases; I_q_, quarantined infected cases; Q, quarantined.

As a result of strict interventional policies taken by Chinese government to control the transmission of SARS-CoV-2, the transmission dynamics had been changed. To decline the infections of COVID-19, plenty of non-pharmaceutical interventions (NPIs), especially social distance strategies, had been implemented [17]. Considering quarantine strategies, models with quarantine compartments were developed. A family of modified SEIR models, named SEIR^+Q^, with quarantine compartments often contains one or more following extension compartments: susceptible people who are quarantined in quarantine center or at home (*S*_*q*_), quarantined individuals in the incubation period (*E*_*q*_) and infected individuals quarantined (*I*_*q*_) [18], [19], [20]. When implementing the social distance strategies, some susceptibles would be required to stay at home for self-quarantine and move to the *S*_*q*_ compartment. In addition, once susceptible individuals contacted infections and became infected, some of them would move to the *E*_*q*_ and *I*_*q*_ compartments once being tracked and quarantined, while some are not quarantined and able to be infected and come into the *E* compartment (Figure 8). Using the SEIR^+Q^ model, Wei et al. estimated that nearly 40% of the total infections was unconfirmed and predicted that on May 31, 2020, the daily new cases in Wuhan would reduce to 0 [18]. Besides, comprehensive interventions and control measures implemented in Wuhan had reduced 19,951 cases by March 30, 2020.

The idea of SEIR^+Q^ model can be easily extended to account for more types of interventions in one single model. For instance, to study the effect of wearing masks, Eikenberry et al. [21] extended SEIR model with a series of compartments representing individuals wearing masks, *S*_*u*_, *E*_*u*_ and *I*_*u*_. Besides, Zhao et al. [22] developed an SEIR model dividing the susceptible people into two compartments, people not taking self-protection actions and people who had taken self-protection actions. Furthermore, to explore possible impact of vaccination, the SEIR models are extended to including a compartment, *S*_*v*_, representing individuals vaccinated. There are evidences that the SARS-Cov-2 virus can survive out of organisms, thus models with a compartment representing virus in the environment were also developed to explore the impact of environment transmission [23, 24]. With different assumptions, these models could explore the possible epidemic trend in the future.

### SEIR model with additional specific parameters

Besides the compartment extensions, parameter extensions are also incorporated to characterize the epidemic in COVID-19. As an example, an indicator for control measure is added to the equation for the change rate of the susceptible population as

$$\frac{dS}{dt}=w\beta IS/N$$

in which *w* represents the impact of control measures on transmission rate, ranging from 0 to 1. Similarly, some studies set transmission rate as a time-varying variable.

$$\frac{dS}{dt}=-\beta \left(t\right)IS/N$$

One important characteristics of SARS-COV-2 virus is that infected individuals in incubation period is infectious. This can be resolved by adding a transmissibility factor to reflect the fact that asymptomatic cases/presymptomatic cases/infections in the incubation period may have different infectivities compared with the transmission rate of symptomatic cases. Thus, for a SEIR model, the transmission from *S* to *I* is now described by following equation

$$\frac{dS}{dt}=-\beta \left(I+\epsilon E\right)S/N$$

While for a SEIAR model with additional compartment of asymptomatic individuals, equation representing the transmission from *S* to *I* is now as followed:

$$\frac{dS}{dt}=-\beta \left(I+\epsilon E+\epsilon A\right)S/N$$

For the SEPIR model, if a presymptomatic compartment was added, the *E* compartment was assumed not infectious and the transmission was shown as the following equation:

$$\frac{dS}{dt}=-\beta \left(I+\epsilon P+\epsilon A\right)S/N$$

where ϵ represent the relative transmissibility factor, ranging from 0 to 1 and often assumed 0.5 or 0.55.

In addition, before the implementation of travel restriction, population flow had a great influence on COVID-19 transmission. Based on mobility data, a series of studies included time-varying parameters into the ordinary differential equations representing the inflow and outflow population in transmission dynamics model. SEIR Models considering population flow are often described by following equations:

$$\left\{ \begin{array}{l} \frac{dS\left(t\right)}{dt}=-\beta I\left(t\right)S\left(t\right)/N+S_{in}\left(t\right)-S_{out}\left(t\right)\\ \frac{dE\left(t\right)}{dt}=\beta I\left(t\right)S\left(t\right)/N-\alpha E\left(t\right)+E_{in}\left(t\right)-E_{out}\left(t\right)\\ \frac{dI\left(t\right)}{dt}=\alpha E\left(t\right)-\gamma I\left(t\right)\\ \frac{dR\left(t\right)}{dt}=\gamma I\left(t\right)\\ N=S\left(t\right)+E\left(t\right)+I\left(t\right)+R\left(t\right)+S_{in}\left(t\right)-S_{out}\left(t\right)+E_{in}\left(t\right)-E_{out}\left(t\right) \end{array}\right.$$

in which *S*_*in*_(*t*) and *E*_*in*_(*t*) represent the numbers of inflow susceptible and exposed individuals at time *t*, respectively, while *S*_*out*_(*t*) and *E*_*out*_(*t*) represent the outflow individuals. In this model, we assume that the infected individuals with symptoms, *I*, would be isolated once they become symptomatic and could not move to other cities.

### Mathematical model with stratified populations

In addition to the compartment extension and parameter extension, another extension to the dynamics model for COVID-19 pandemic is the population-stratified compartment model. While most transmission dynamics models, such as SIR/SEIR model and their extensions, have been successfully used for small communities or cities but failed to capture the dynamics at larger area, and lacked the ability to account for the interactions between metapopulations via mixing/commuting [25]. Furthermore, the substructure of a population due to different demographic characteristics, such as age, gender and occupation, etc., lead to the heterogeneities on the susceptibility to infection, contact rate, fatality rate within a population, and so on. Thus, population-stratified models, also called metapopulation models, were developed by dividing the population into a series of smaller units according to geographic location or demographic structure [13]. As an example, a metapopulation model including information of geographic location is shown as follows:

$$\left\{ \begin{array}{l} \frac{dS_{i}\left(t\right)}{dt}=-\beta I_{i}\left(t\right)S_{i}\left(t\right)/N_{i}\left(t\right)+\sum_{j=1,i\neq j}^{n}M_{ji}\left(t\right)\frac{S_{j}\left(t\right)}{N_{j}\left(t\right)-I_{j}\left(t\right)}-\sum_{j=1,i\neq j}^{n}M_{ij}\left(t\right)\frac{S_{i}\left(t\right)}{N_{i}\left(t\right)-I_{j}\left(t\right)}\\ \frac{dE_{i}\left(t\right)}{dt}=\beta I_{i}\left(t\right)S_{i}\left(t\right)/N_{i}\left(t\right)-\alpha E_{i}\left(t\right)+\sum_{j=1,i\neq j}^{n}M_{ji}\left(t\right)\frac{E_{j}\left(t\right)}{N_{j}\left(t\right)-I_{j}\left(t\right)}-\sum_{j=1,i\neq j}^{n}M_{ij}\frac{E_{i}\left(t\right)}{N_{i}\left(t\right)-I_{j}\left(t\right)}\\ \frac{dI_{i}\left(t\right)}{dt}=\alpha E_{i}\left(t\right)-\gamma I_{i}\left(t\right)\\ \frac{dR_{i}\left(t\right)}{dt}=\gamma I_{i}\left(t\right)+\sum_{j=1,i\neq j}^{n}M_{ji}\left(t\right)\frac{R_{j}\left(t\right)}{N_{j}\left(t\right)-I_{j}\left(t\right)}-\sum_{j=1,i\neq j}^{n}M_{ij}\left(t\right)\frac{R_{i}\left(t\right)}{N_{i}\left(t\right)-I_{j}\left(t\right)}\\ N_{i}\left(t\right)=S_{i}\left(t\right)+E_{i}\left(t\right)+I_{i}\left(t\right)+R_{i}\left(t\right)+\sum_{j=1,i\neq j}^{n}M_{ji}\left(t\right)-\sum_{j=1,i\neq j}^{n}M_{ij} \end{array}\right.$$

*S*_*i*_(*t*), *E*_*i*_(*t*), *I*_*i*_(*t*) and *R*_*i*_(*t*) denote the number of individuals belonging to the *S*, *E*, *I* and *R* compartments of city *i* at time *t*. *M*_*ji*_(*t*) represents the number of individuals moving from city *j* to *i* at time *t* and vice versa. In this model, the infected individuals with symptom are also assumed to be isolated.

Besides, an age-structured SEIR model is shown as follows:

$$\left\{ \begin{array}{l} \frac{dS_{i}\left(t\right)}{dt}=-\Lambda_{i}S_{i}\left(t\right)/N\left(t\right)\\ \frac{dE_{i}\left(t\right)}{dt}=\Lambda_{i}S_{i}\left(t\right)/N\left(t\right)-\alpha E_{i}\left(t\right)\\ \frac{dI_{i}\left(t\right)}{dt}=\alpha E_{i}\left(t\right)-\gamma I\left(t\right)\\ \frac{dR_{i}\left(t\right)}{dt}=\gamma I_{i}\left(t\right)\\ \Lambda_{i}=\sum_{j}\beta_{ji}I_{j}\left(t\right)\\ N\left(t\right)=\sum_{i}\left(S_{i}\left(t\right)+E_{i}\left(t\right)+I_{i}\left(t\right)+R_{i}\left(t\right)\right) \end{array}\right.$$

in which *S*_*i*_(*t*), *E*_*i*_(*t*), *I*_*i*_(*t*) and *R*_*i*_(*t*) denote the number of individuals in age group *i* at time *t*; *β*_*ji*_ represents the transmission rate from age group *j* to *i*, which would change along with the contact rate between age group *i* and *j*.

## Applications of transmission dynamic models in the pandemic of COVID-19

As of July 05, 2021, we identified 1095 articles on PubMed by using a keyword of “((COVID-2019) OR (ncov-2019) OR (COVID-19) OR (2019-nCoV) OR (novel coronavirus) OR (SARS-CoV-2)) AND ((transmission dynamics model) OR (compartment model))”. Applications of transmission dynamics models could be categorized into the following classes: epidemiological parameter estimation, trend prediction, control measure evaluation and possibility exploration (Table 2).

**Table 2:** Applications of transmission dynamics models in COVID-19.

Objectives	
Epidemiological parameter estimation	–
Trend prediction	Predicting confirmed cases
	Predicting hospitalized cases
	Predicting deaths
Control measure evaluation	Reduction on transmission
	Reduction on cumulated cases
	Reduction on hospitalizations
Possibility exploration	Simulating different assumptions about interventions
	Exploring long-term transmission dynamics in different assumptions
	Simulating different assumptions of vaccination
	Exploring the start time of epidemic

### Estimation of infectious parameters at early stage of the COVID-19 epidemic

A key parameter in transmission dynamics models is the basic reproductive number (*R*_0_), which representing the average number of next generation cases infected by one infected individual introduced into the population in which all people are assumed susceptible [1]. The effective reproductive number, *R*_*e*_, or time-varying reproductive number, *R*_*t*_, is defined to be the average number of next generation cases produced by one infected individual during the infectious period, which would change by time t after the initial infected cases introduced [26].

At the beginning of SARS-CoV-2 epidemic, knowledge of the epidemiologic characteristics and the virus is limited. Transmission dynamics model can be used derive the epidemiologic characteristics and biologic features, such as *R*_0_, epidemic doubling time and the infectiousness of asymptomatic infections.

Published on January 31, 2020, just 8 days after the “lockdown” of Wuhan city, Wu et al. used a modified SEIR model using the reported data before Jan 25, 2020, as well as population flow information. The model they used also included some parameters obtained through prior information and experiences. They estimated that 75,815 people (572 reported) that had been infected in Greater Wuhan area before Jan 25, 2020, and warned that it is possible that several large cities, including Beijing, Shanghai, Guangzhou, Shenzhen and so on, had imported hundreds of infections from Wuhan before the “lockdown” [27]. The ordinary differential equations they used are as following:

$$\left\{ \begin{array}{c} \frac{dS\left(t\right)}{dt}=\frac{S\left(t\right)}{N}\left(\frac{R_{0}}{D_{I}}I\left(t\right)+z\left(t\right)\right)+L_{I,W}+L_{C,W}\left(t\right)-\left(\frac{L_{W,I}}{N}+\frac{L_{W,C}\left(t\right)}{N}\right)S\left(t\right)\\ \frac{dE\left(t\right)}{dt}=\frac{S\left(t\right)}{N}\left(\frac{R_{0}}{D_{I}}I\left(t\right)+z\left(t\right)\right)-\frac{E\left(t\right)}{D_{E}}-\left(\frac{L_{W,I}}{N}+\frac{L_{W,C}\left(t\right)}{N}\right)E\left(t\right)\\ \frac{dI\left(t\right)}{dt}=\frac{E\left(t\right)}{D_{E}}-\frac{I\left(t\right)}{D_{I}}-\left(\frac{L_{W,I}}{N}+\frac{L_{W,C}\left(t\right)}{N}\right)I\left(t\right) \end{array}\right.$$

In the above equations, *S*(*t*), *E*(*t*) and *I*(*t*) were the number of susceptible, exposed and infected individuals at time *t*; *D*_*E*_ and *D*_*I*_ were the average incubation period (assumed 6 days according to that of SARS-CoV and MERS-CoV) and infectious period (the serial interval minus the average incubation period, 8.4 days–6 days = 2.4 days) [28]; *L*_*W*,*I*_ is the daily average number of international outbound air passengers from Wuhan, while *L*_*I*,*W*_ is that of international inbound air passengers; *L*_*W*,*C*_ is the daily number of all domestic outbound travelers from Wuhan and *L*_*C*,*W*_ is that of all domestic inbound travelers; *R*_0_ divide *D*_*I*_ represent the transmission rate; *z*(*t*) was the zoonotic force of infection. In this model, *R*_0_ was estimated as 2.68 (95% CI: 2.47–2.86), while the epidemic doubling time was 6.4 days (95% CI: 5.8–7.1). In addition, other early studies had estimated *R*_0_ of COVID-19 in Italy before February 22, 2020 and in Germany before March 15, 2020 were 3.6 (95% CI: 3.49–3.84) and 3.4 (95% CI: 2.4–4.7) respectively [29, 30].

At the early stage of the epidemic in China, although the infectiousness of asymptomatic or mild symptomatic cases had been proved, the infectiousness and proportion of these infections in China and how they would contribute to the spread of COVID-19 remains unknown [31], [32], [33]. Thus, Li et al. combined the mobility data, and developed a metapopulation model stratified population by geographic units and simulating spatio-temporal dynamics of COVID-19 among 375 Chinese cities, using the following equations:

$$\left\{ \begin{array}{c} \frac{dS_{i}}{dt}=-\frac{\beta I_{i}^{r}S_{i}}{N_{i}}-\frac{\mu \beta I_{i}^{u}S_{i}}{N_{i}}+\theta \sum_{j=1,i\neq j}^{n}M_{ij}\frac{S_{j}}{N_{j}-I_{i}^{r}}-\theta \sum_{j=1,i\neq j}^{n}M_{ji}\frac{S_{i}}{N_{i}-I_{i}^{r}}\\ \frac{dE_{i}}{dt}=\frac{\beta I_{i}^{r}S_{i}}{N_{i}}-\frac{\mu \beta I_{i}^{u}S_{i}}{N_{i}}-\frac{E_{i}}{Z}+\theta \sum_{j=1,i\neq j}^{n}M_{ij}\frac{E_{j}}{N_{j}-I_{i}^{r}}-\theta \sum_{j=1,i\neq j}^{n}M_{ji}\frac{E_{i}}{N_{i}-I_{i}^{r}}\\ \frac{dS_{i}^{r}}{dt}=\alpha \frac{E_{i}}{Z}-\frac{I_{i}^{r}}{D}\\ \frac{dS_{i}^{u}}{dt}=\left(1-\alpha \right)\frac{E_{i}}{Z}-\frac{I_{i}^{r}}{D}+\theta \sum_{j=1,i\neq j}^{n}M_{ij}\frac{I_{i}^{r}}{N_{j}-I_{i}^{r}}-\theta \sum_{j=1,i\neq j}^{n}M_{ji}\frac{I_{i}^{r}}{N_{i}-I_{i}^{r}}\\ N_{i}=N_{i}+\theta \sum_{j=1,i\neq j}^{n}M_{ij}=\theta \sum_{j=1,i\neq j}^{n}M_{ji} \end{array}\right.$$

in which $S_{i},E_{i},I_{i}^{r},I_{i}^{u}\text{ and }N_{i}$ are the susceptible, exposed, documented infected, undocumented infected and total population in city *i* [13]. In this model, $I_{i}^{u}$ and $I_{i}^{r}$ represents infected individuals who are asymptomatic or mildly symptomatic and unreported and individuals whose symptoms are severe enough to be detected. Similar to extension of SEIR models we mentioned at Section 3.3, the transmission rate of undetected individuals is reduced by a factor *μ*, the same as ϵ in Section 3.3. *M*_*ij*_ denotes the daily number of people traveling from city *j* to city *i*. To correct for underreporting of human movement, a multiplicative factor *θ*, greater than 1, is included in the equations. Individuals in the compartment are assumed to be isolated and cannot move between cities. In conclusion, this model is a combination of three different extensions, a compartment extension (*I* ^*u*^), a parameter extension (*μ* the same as ϵ ) and geographic units.

Indeed, Li’s model not only included additional compartment for unascertained cases, but also incorporating additional parameters for transmission and sub-populations, as we have introduced above. According to this study, *R*_*e*_ at the beginning of the epidemic (10–23 Jam, 2020), the same as *R*_0_, is 2.38 (95% CI: 2.03–2.77) and undetected infections, had 55% transmission rate (*μ*) compared with detected infections (95% CI: 46%–62%). Besides, this model also estimated the median the incubation and infectious periods are 3.69 and 3.47 days, respectively, providing references for further studies; and only 14% (95% CI: 10%–18%) infections in mainland China were detected and reported during January 10–23, 2020.

As a large number of infected individuals but without or with mild symptoms were unascertained, picturing the dynamics of COVID-19 during the early stage is critical for making monitoring and control strategies, assessing the possibility for future transmission in areas which imported several cases, as well as early warning and prevention of possible COVID-19 outbreaks. Kucharski et al. [34] constructed an SEIR model with three more compartments, asymptomatic cases, symptomatic but unreported cases and confirmed cases, and considering international travel. The study found that time-varying *R*_*t*_ in Wuhan was 2.35 (95% CI 1.15–4.77) on January 16 and declined to 1.05 (95% CI: 0.41–2.39) on January 31. Besides, they also estimated that once four or more infected individuals came into one location, the epidemic would potentially begin.

### Trend prediction

With increasing knowledge of SARS-CoV-2, a series of models were proposed to predict the future trend of the COVID-19 epidemic. However, the prediction of the “exact” number of cases is impossible due to limited information of the virus, the disease, the environment and the population. As an example, we will never know how many people are, or had been, infected actually. The powerful control measures, as well as the improved health habits (more frequent to wash hands, wearing masks in public areas, etc.) of people, make the predictions even more difficult [35]. Thus, we believe that the value of predictions made by transmission dynamic models is to provide possible trend given the current epidemic situations and future interventional policies and measures. Predictions cannot tell us what must happen in the future but can warn us what will happen if something had been/not been changed.

#### Predictions on the number of confirmed cases

**Figure 9:**
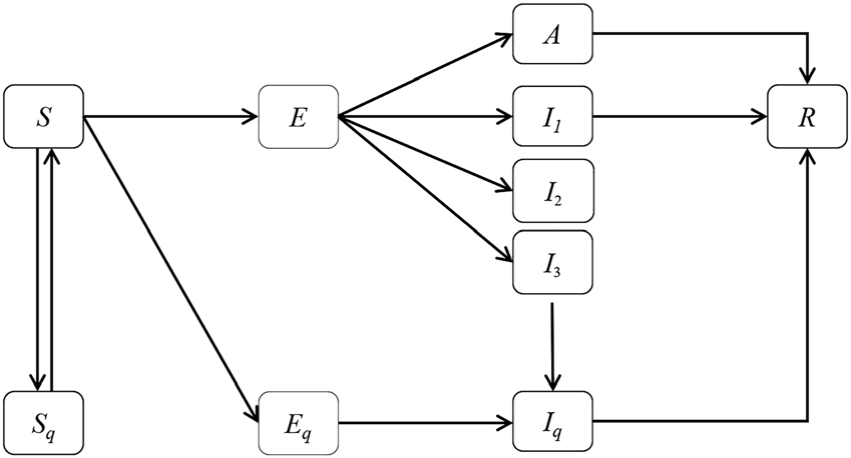
Diagram of SEIR + CAQ model. S, susceptible; E, exposed; I_1_, infected individuals with mild symptom; I_2_, infected individuals with severe symptom; I_3_, infected individuals with critical symptom; R, recovered; S_q_, quarantined susceptible; E_q_, quarantined latent cases; I_q_, quarantined infected cases; C, infected components; A, asymptomatic infected; Q, quarantined.

Using data up to February 12, 2020, Wei et al. modeled the cumulated confirmed cases in three regions, Wuhan, Hubei except Wuhan and China except Hubei, by using a SEIR^+CAQ^ model (SEIR with Infected Components, Asymptomatic infected and Quarantined individuals, Figure 9) [36]. Their results showed that the pandemic in China except Hubei and Hubei except Wuhan had reached the peak on February 1, 2020 or February 2, 2020, while the peak time of Wuhan was February 9, 2020. They predicted that under prevention and control measures during the model was constructed, there would be 80,417 (actually 79,215) accumulated confirmed cases in China up to February 29, 2020.

Yang et al. [37] modified the SEIR model by adding a move-in and a move-out compartments. They predicted that there would be a peak on February 20, 2020 with cumulative active infections of 42,792 (95% CI: 30,149–52,941), and the end of the pandemic is expected to be in late April, 2020, with totally 59,578 (95% CI: 39,189–66,591) cases in Hubei province. Besides, the study also predicted the peaks of accumulated active infections of Guangdong province and Zhejiang province would be 1,202 (95% CI: 1,042–1,340) and 1,172 (95% CI: 1,004–1,314) by February 20, 2020, while the accumulated active infections in mainland China will reach the peak at 59,764 (95% CI: 51,979–70,172) on February 28, 2020. Furthermore, based on the predictions, the cumulated cases in Hubei province, Guangdong province, Zhejiang province and mainland would finally reach 59,578 (95% CI: 39,189–66,591), 1,511 (95% CI: 1,097–1,948), 1491 (95% CI: 1,066–1,851) and 122,122 (95% CI: 89,741–156,794).

Wang et al. [38] proposed a SEIQRD model with three compartments for quarantined individuals and an additional compartment for deaths, to model the trend of epidemic using the cumulative confirmed cases between January 10 and February 4, 2020 in China. They predicted that the pandemic in mainland China would reach its peak around February 11 with a peak size of 4,066 (95% CI: 3898–4472) daily confirmed cases, and reach the end around May 18, 2020.

At the early stage of pandemic, a large number of modeling studies were focused on epidemic in China. After outbreaks of SARS-CoV-2 in countries beside China, a series of predictive studies on future epidemic trend in these countries were also reported. According to Gatto’s study, up to March 25, 2020, the cumulative number of infections in Italy was approximately 733,000 individuals, while Fanelli and Piazza reported that the peak number of active confirmed cases in Italy would be about 26,000 around March 21, 2020 [29, 39].

Besides, compartment models with various extensions, such as compartment of unascertained infections [40, 41], virus in the environment [24], quarantine [41], [42], [43], [44], [45], compartment of taking infection prevention actions [22], the effect of control measures [46], as well as other extensions [47], [48], [49], [50], were constructed and make contribution on the early warning of COVID-19 spread.

#### Predictions on the number of hospitalized cases

Transmission dynamics model could also be used to predict the patients in hospital so as to provide information about the demand for medical resources. By using a modified SEIR model incorporating additional compartments of individuals in different disease stages, quarantine, hospital and ICU, as well as considering different transmission rates associated with different ages, Tuite et al. predicted that in Ontario, Canada, 56% (95% CI: 42%–63%) individuals would be infected. Their model also predicted that, in Ontario, Canada, there would be 107,000 (95% CI: 60,760–149,000) cases in hospital (non-ICU) and 55,500 (95% CI: 32,700–75,200) cases in ICU at the peak time [51]. To predict the demand of ICU bed and future hospital occupancy in Switzerland, Zhao et al. [52] built a platform based on two different SEIR model and simulated hospital occupancy under a series of epidemic scenarios. Similarly, Verma et al. [53] developed a modified age-structure SEIR model to predict future demand for hospital resources in India.

#### Predictions on death toll

Transmission dynamic models with a compartment of deaths are also able to predict the number of deaths. Based on data in Italy up to May 15, 2020, Fanelli et al. [39] developed the SIRD model, an extension of SIR model with a compartment of dead individuals (D), and predicted that the cumulated number of deaths would be about 18,000 in the final. Besides, Cui et al. predicted that the final size of deaths in Wuhan would be 3,206 by a modified SEIR model incorporating quarantine strategies. In addition, based on SIHRD model, SIR model with hospitalized (H) and dead (D) compartment, Cuadros et al. estimated that there would be 1,421 deaths in Ohio, US, as of May 10, 2020 [54].

### Policy evaluation

To control the transmission of COVID-19, governments have taken kinds of intensive non-pharmaceutical interventions. In order to take effective and prompt measures in potential future epidemics, it is important to evaluate how the interventions strategies influence the transmission of COVID-19 [55]. Transmission dynamics models can be applied to estimate the effectiveness of non-pharmaceutical interventions, such as travel restrictions between cities, social distancing measures inner cities. The effects of control measures are often estimated by comparing values of transmission parameters (contact rate, transmission rate, *R*_*e*_ and *R*_*t*_, etc.) before introducing the interventions to that after introducing the interventions, or comparing the observed numbers (cases, hospitalizations and death, etc.) to the expected number without the interventions.

#### Evaluation on the effect of blocking transmission

On January 23, 2020, after confirming transmission of COVID-19 between human, Chinese government immediately implemented unprecedented cordon sanitaire in Wuhan. All transportation paths to and from Wuhan had been shut down. Later, public health “level 1” emergency response was implemented all over China [56].

To evaluate the effect of “level 1” emergency response in China cities and Wuhan travel ban, Tian et al. added a parameter on control effect in a SEIR model. Based on Tian’s study, *R*_0_ in Wuhan before January 23 was 3.15 (95% CI: 3.04–3.26), and reported that the implementation of “level 1” emergency response and Wuhan travel ban had reduced 3%–69% transmission in provinces except Hubei and avoided 96% new cases (nearly 450,000) outside Wuhan up to February 19, 2020 [57].

Tang et al. reported that, adopting the most severe prevention and control measures and highest level of detection and treatment, the contact rate would have a reduction of 80.2%, decreasing from 14.781 to 2.9253 [19]. By assessing the effect of control measures on reduction of contact rate, Tang et al. estimated that public health measures taken in Ontario, Canada since February 26, 2020 had reduced 80.1% contact rate, from 11.58 to 2.20 [58]. Besides, the change of *R*_*e*_ was also widely used to assess the effect of control strategies [57–67]. Similarly, the reduction of transmission rate can also indicate the effect of interventions [62, 68], [69], [70], [71], [72], [73], [74].

On February 12, 2020, an updated clinical diagnostic criteria—patients without positive nucleic acid test could be diagnosed as confirmed cases if they have clinical and radiological characteristics typical of COVID-19—was implemented in Hubei Province, resulting in a rapid increment of cases in Wuhan in short time [75]. In addition, a second round of universal symptom survey was taken during February 17 to 19, 2020. Under this condition, Wei et al. extended SEIR transmission dynamics model by considering three quarantined compartments (SEIR^+Q^) to evaluate the effects of these major interventions [18]. The effect of control measures can be estimated by comparing the expected trend with observed trend of cumulated clinical confirmed cases. According to this study, nearly 40% infected cases was unconfirmed before February 12, 2020 and the updated clinical diagnostic criteria and two universal symptom surveys had reduced 19,951 cases by March 30, 2020.

#### Evaluation on the effect of reducing cases

Comparing the trend of cumulated cases with and without implementing intervention and control measures is a widely used method to evaluate the effect of these control measures [69, 76], [77], [78], [79], [80]. To picture the COVID-19 dynamics in Wuhan, Hao et al. extended the SEIR model with three additional compartment, presymptomatic infectiousness (*P*), unascertained cases with no or mild symptoms (*A*) and case in hospital (*H*), and modeled the outbreak divided into five periods according to different degrees of interventions from January 1, 2020 [12]. The estimated *R*_0_ was 3.54 (3.40–3.67) and the ascertained rate of infections in Wuhan before January 23, 2020 was 0.15 (0.13–0.17). The public health interventions had reduced 92% transmission since January 23, 2020. If no interventions had been implemented in Wuhan, the total number of infections would be 6,302,694 (6,275,508–6,327,520) on March 8, 2020. By comparing the expected number to that observed (49,912), the effect of control measure was estimated.

Besides, quarantine strategies had also contributed a lot to control the COVID-19 transmission. Zu et al. [81] based on a dynamics model reported that, since January 23, 2020, the quarantine measures in mainland China had avoided 99.85% total confirmed cases and 99.84% deaths.

#### Evaluation the effect on reduction on hospitalizations

Gatto et al. [29] proposed an extended SEIR model with presymptomatic, hospitalized, asymptomatic/mildly symptomatic and quarantined compartments to assess the effectiveness of emergency containment measures initiated in Italy. According to their study, containment measures in February had avoided 226,000 hospitalizations (95% CI: 172,000–347,000) as of March 25, 2020. Furthermore, the implementation of all containment measures in Italy had reduced 45% (95% CI: 42%–49%) transmission and avoided 6,490,000 hospitalizations (95% CI: 4,810,000–10,100,000) as of March 25, 2020.

### Forecasting of future possibilities

Transmission dynamics models based on SIR/SEIR frameworks are able to simulate various transmission trends under different settings so as to guide future policies. With better understanding of SARS-CoV-2 characteristics and the influence of interventions estimated by models, it is logical and reasonable to make simulation on the possibilities in the future. Disease-specific or intervention-specific parameters in transmission dynamics models can be modified based on knowledge of underlying transmission process to explore what would happen under different assumptions of disease characteristics and interventions [35]. At early dynamics, biological features and epidemiological characteristics of SARS-CoV-2 had not been measured and dynamics models were used to explore the possible value of this parameter, such as the latent period, the relative transmission rate of exposed, presymptomatic or asymptomatic infections, the duration of infectiousness, generally represented by an estimation with a range, reflecting uncertainty. Estimating the unknown parameters can provide references for experiments. Predicting the future trend of epidemic is also a form of exploring possibility by simulating the possible future trend conditional on current measures and parameters [35]. Besides, when assessing the control measures, comparing the trend with interventions (observed) with the trend without interventions (expected), is a commonly used method to measure the effect of interventions.

#### Simulating different assumptions about interventions

In SIR/SEIR models and their extensions, interventions can be included by adding additional compartments or transmission parameters, which makes it possible for the models to be uses to explore and estimate the influence of different assumptions of interventions. Using transmission dynamics model, scientists can not only make prediction on what would happen if a control measure were or were not taken, but also understand how a change in intervention would affect the epidemic by assuming different effect size and implementing time of interventions. Further, as many studies have already reported the effect of control measure in transmission which had been implemented, scientists can then understand what would happen if control measures were lifted and how to control the epidemic in a long term without damaging economy and health systems.

##### Different effect of intervention

Based on the fitted transmission dynamic models in one place, we can explore the possibility of outbreaks in other cities and whether the outbreak can be controlled and how to achieve. Kucharski et al. [34] found that a city will have a probability of 50% of COVID-19 outbreak if at least four cases were imported. By assuming different effect of quarantine strategies implemented in Wuhan on inter-city and inner-city mobility reduction on Jan 23, 2020, Wu’s simulation indicated that the influence of inter-city mobility reduction on COVID-19 transmission was negligible, while inner-city mobility reduction could delay the peak of confirmed cases, flatten the curve and reduce the magnitude [27]. Zhao et al. [82] simulated the future transmission by an improved SEIR model with different assumed effect of interventions and emphasized the importance of control strength, control time and an awareness of self-protection, such as facemask and wash hands. Other studies had also explored the future epidemic with assumed effect of interventions or different quarantined proportion of exposed individuals [20, 22, 39, 42, 83], [84], [85], [86], [87], [88], [89], [90], [91], [92], [93], [94], [95]. Wang et al. simulated the trend of cumulated deaths with assumed R_e_ and death rate in different stages of control [96].

##### Different time of intervention

The implementing time of intervention and control measures had a great influence on transmission of COVID-19 and, by assuming different time introducing an intervention, transmission dynamics models could help understand what would happen when implementing the intervention at different time. According to Yang et al., 5 days later the introduction of interventions was, there would be a huge increase in transmission rate and the total epidemic size by the end of April will be 351,874 cases, while total epidemic size would have been only 40,991 if 5 days earlier the interventions were introduced, suggesting that the importance of implementation time of containment measures [37]. Assuming implementing different interventions in Germany, Dehning et al. divided the epidemic into several stages according to interventions and simulated the possible future trend under diverse control scenarios [30]. Similar to Yang et al., Dehning et al. reported that the timepoint of introducing social distance strategies been advanced or delayed by just five days would cause a three times difference at least in cumulated cases.

In New York, US, a series of social distance strategies had been introduced since March 12, 2020. According to Alagoz’s study, in which COVAM, an extension of SEIR considering the stages of COVID-19 and age structure, was constructed to explore the effect of social distancing measures [97]. One week earlier social distancing measures were introduced, the total number of cases in New York city would reduce from 203,261 to 41,366 up to May 31, 2020, while the number of confirmed cases would increase to 1,407,600 if interventions were implemented 1 week later.

Different start time of lockdown and quarantine is also significantly important for the disease controlling and a series of studies had proved that the delay of lockdown and quarantine strategies would lead to catastrophic results [42, 65, 74, 79, 93, 98, 99]. Besides, Wang et al. simulated the scenarios assuming different opening time of the “Huoshenshan” hospital, a quarantine hospital built in 9 days, and found that delayed opening the “Huoshenshan” hospital would greatly increase the magnitude of the outbreak by about 80.1% higher than the real data by March 6, 2020 [99].

Rong et al. and Zu et al. simulated delay in diagnosis would have great influence on the disease transmission based on new dynamics models [23, 81]. Besides, a few studies indicated the more media reports of COVID-19, the slower the spread of COVID-19, possibly as a result of increasing awareness of self-protection caused by media reports [100], [101], [102].

##### Exploring feasible dynamic intervention strategy

Governments have taken different interventional policies to combat against the pandemic of COVID-19. While strict control strategies are essential to slowing down the transmission of virus in a short term, the negative impacts of these strategies, including economic risks, increasing of unemployment, health system collapse, etc., have become to emerge in the long-term [103]. In this situation, transmission dynamics models could be applied to explore the possible consequences of different decisions before they were implemented and optimize the comprehensive strategies balancing the preventions and control measures and economy severely damaged by sustained and strict interventions. As an example, the relaxation of social distance strategies may have the risk of the resurgence of the epidemic. Aleta et al. [104] proposed a response system based on dynamics model with compartments of different stages of infection and indicated that, to control the transmission, lifting social-distancing interventions would require high ability of contact tracing and testing. To explore a more feasible intervention strategy, Tuite et al. developed a dynamic interventions strategy which can be introduced and lifted in response to real-time state of the epidemic. Their simulations indicated that the dynamic interventions strategy is an effective and more feasible control strategy [51].

While NPIs could effectively reduce the infections and prevent the overburden of the health systems, these prolonged suppression measures are unsustainable and have adverse influence on economy in most countries. Thus, understanding how and when to relax the NPIs so as to minimize the damage to the economics while keep the epidemic under control becomes an important issue. Chowdhury et al. [16] modelled the impacts of different types of interventions on ICU admissions and the number of deaths in 16 worldwide countries. They proposed that a dynamic cycles of 50-day strong interventions, keeping *R*_*t*_ below 0.5, and then 30-day relaxation could sufficiently keep ICU demands well under the national capacities for all countries. Further, another study also proved the efficiency of “on-off” policies alternating between the introduction and relaxation of strict social distancing [105]. In addition, Kennedy et al. also modeled 48 possible future scenarios considering several social-distance strategies including mitigation, stepping-down strategy, intermittent strategy and constant strategy [106]. They proposed a stepping-down approach every 80 days which could most effectively reduce the transmission over a two-year period, prevent a second outbreak and keep the number of ICU demands per day well under the threshold of current capacities.

##### Explore the possibility and strategy of reopening

In May, 2020, the prevention and control measures of COVID-19 in Canada had achieved initial success. Whether it is appropriate to relax some distancing measure and resume economic activities becomes a new consideration. Tang et al. [58] performed simulations on de-escalation of control measures in Ontario, Canada and explored the possibility of reopening. They warned that the less social distance strategies maintained, the more exposed contacts (maybe unrealistically high) should be effectively tracked and isolated to keep *R*_*t*_<1. Another study had simulated lifting social-distancing requirements in the US and obtained similar conclusion [107]. Di Domenico et al. [108] evaluated the impact of lift strategies in COVID-19 transmission in Île-de-France by an age-structured SEIR model with compartments of severity of diseases, hospitalized and ICU. A similar study for epidemic in India simulated the trend after removing lockdown [109]. Several studies had also explored the possible trend of reopening social distance strategies [54, 81, 91, 110], [111], [112], [113], [114].

##### Exploring the influence of wearing masks on COVID-19 epidemic

Masks are widely used to protect the susceptibles. However, there is still controversy on whether to recommend to wearing masks for general public in some western countries. Transmission dynamics models with a compartment representing individuals or a parameter of the effect of masks on transmission had been developed to estimate how the masks can protect us from COVID-19. According to Dai’s study, only extremely strong interventions (reducing 90% transmission of COVID-19, equal to all people wearing N95 respirators) could sufficiently prevent the transmission of COVID-19 [77]. Eikenberry et al. [21] designed a modified SEIR model with several compartments of individuals wear masks in public. In their study, for regions where the transmission rate was high, such as New York, 17%–45% deaths over two months could be prevented and there would be a 34%–58% decrease on peak daily death rate, if 80% individuals wear moderately (50%) effective masks, even there are no other changes in epidemic dynamics. Besides, for regions with low or decreasing transmission rate, very weakly (20%) effective masks also have value to some extent on prevention. Similarly, masks have also contributed to avoid deaths. Worby and Chang based on a modified SEIR model with three compartments of presymptomatic cases, asymptomatic cases and symptomatic cases simulated the scenarios of different supply and demand of medical resources. When the medical resources are shortage, providing masks with high effect to infected individuals and the elder could achieve the optimal outcome of deaths. They also reported that wearing masks, even homemade facemasks offering 5% protection, universally in public could cause a 10% reduction on *R*_*t*_ and a 3%–5% reduction in deaths [115]. Other studies had also proved the effect of wearing masks using various transmission dynamics models [116].

##### Exploring feasibility of novel control strategy

Transmission dynamics model can also be used to explore the feasibility of a new control strategy. Weitz et al. [117] proposed a new approach, named shield immunity, which can be implemented together with social distancing, to limit transmission. The core concept of the shield immunity is to deploy the recovered individuals who have immunity to SARS-CoV-2 back into the community, and these individuals substitute susceptible individuals to contact with infectious individuals, relatively lowering the contact rate of susceptible individuals and infections. They conducted simulations using a modified SEIR model in different transmission scenarios and provided evidences on the effectiveness of shielding. Furthermore, another study by Kabir et al. [118] combined theories of transmission dynamics models with the concept of behavioral dynamics and proved the feasibility and availability of shield immunity. Besides, reducing the possibility of superspreading events was also a considerable and valuable option to alleviate the need for strict social distancing strategies [119]. To reduce transmission of COVID-19 and hospital burden, Reimer proposed Clinical prediction rules using five clinical variables to identify individuals who would be most possibly infected by SARS-CoV-2 and, with the help of a stochastic SEIR compartmental model of transmission, they confirmed the effectiveness of those rules on delaying the peak time and reducing the peak number and final number of infections over the pandemic [120].

To mitigate the impact of the limited medical resources, Upadhyay et al. [67] proposed a strategy of age-targeted testing, deploying more testing resources on the most possibly vulnerable age groups. Based on an age-structured SIQR model, SIR model with a quarantine compartment, they found that, in India, increasing testing in the 15–40 years age group, the most infected age group, could help efficiently reduce the infected cases. Similarly, Wilder et al. [121] reported that targeted shelter for 50% individuals in a single age group may substantially lower the transmission, and they also highlighted the role of between-population variation in formulating interventions. In addition, there are studies using compartment models to explore optimal control designs [122], [123], [124], [125], [126], [127], [128], [129].

Some countries were aiming for herd immunity, such as Sweden and the United Kingdom. Brett and Rohani [130] built an age-structured SEIR model for UK to explore the prerequisite conditions of herd immunity. According to their study, to achieve herd immunity, social distance should be reduced at the exact rate to re-increase the transmission and, in this case, the proportion of individuals who have immunity against SARS-CoV-2 would increase. Herd immunity required that social distance which is influenced by NPIs needs to be gradually lifted and highly controlled in a long period—too quickly the NPIs are lifted, the medical system and medical resources would face great challenges; too slowly, the epidemic would come to an end without achieving herd immunity. Therefore, complete herd immunity is possibly impractical.

#### Exploring long-term transmission dynamics

To explore the future of COVID-19 transmission, Kissler et al. [14] simulated the long-term trend of the COVID-19 epidemic based on a modified SEIR model considering meteorological factor, immunity, and cross-immunity for beta-coronaviruses OC43 and HKU1. According to the study, the recurrent outbreaks of SARS-CoV-2 is highly seasonal variated. Depending on the duration of immunity, SARS-CoV-2 dynamics in the future would occur once a year, once two years, sporadically, which means the pandemic would enter into seasonally regular circulation, or, only if the immunity to SARS-CoV-2 is permanent, never. Besides, they reported social distance strategies may be necessary up to the end of 2022 to prevent critical care capacity from being overburden.

#### Simulating different outcomes after vaccination

**Figure 10:**
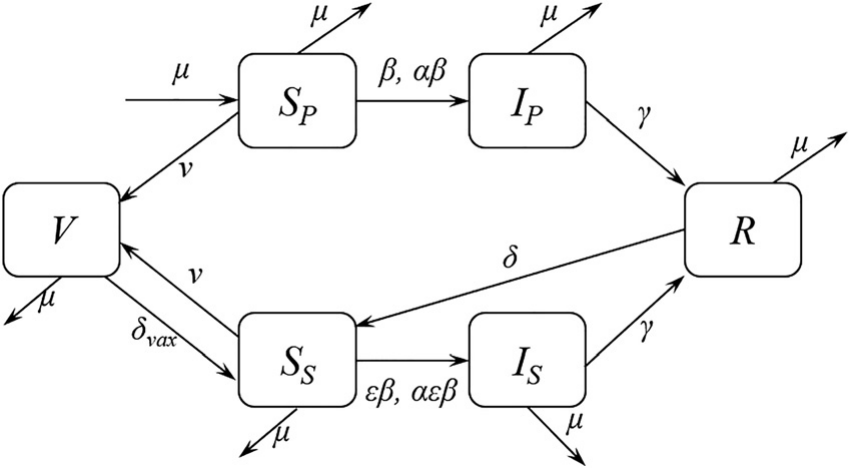
Modified model flowchart that incorporates a vaccinated class V and reinfection classes. S_P_, fully susceptible individuals; S_S_, partially immune individuals; R, fully immune individuals; V, vaccinated individuals; I_P_, primary infected cases; I_S_, secondary infected.

Vaccination is one of the key methods to terminate the pandemic of COVID-19, makes it important to make sense of how vaccine would influence the dynamics of COVID-19 in the future. Saad-Roy et al. [131] developed a modified model SIR model with additional compartments representing individuals who have immunity (Figure 10) for a series of immune scenarios to explore futures for COVID-19 transmission with and without vaccines. The ODEs of Saad-Roy’s model are as following:

(16) $$\left\{ \begin{array}{c} \frac{dS_{P}}{dt}=\mu -\beta \left(t\right)S_{P}\left(I_{P}+\alpha I_{S}\right)-\mu S_{P}-s_{vax}\nu S_{P}\\ \frac{dI_{P}}{dt}=\beta \left(t\right)S_{P}\left(I_{P}+\alpha I_{S}\right)-\left(\gamma +\mu \right)I_{P}\\ \frac{dR}{dt}=\gamma \left(I_{P}+I_{S}\right)-\delta R-\mu R\\ \frac{dS_{S}}{dt}=\delta R+\delta_{vax}V-\epsilon \beta \left(t\right)S_{S}\left(I_{P}+\alpha I_{S}\right)-\mu S_{S}-s_{vax}\nu S_{S}\\ \frac{dI_{S}}{dt}=\epsilon \beta \left(t\right)S_{S}\left(I_{P}+\alpha I_{S}\right)-\left(\gamma +\mu \right)I_{S}\\ \frac{dV}{dt}=s_{vax}\nu \left(S_{P}+S_{S}\right)-\left(\delta_{vax}+\mu \right)V \end{array}\right.$$

In this model, six compartments are included: fully susceptible, *S*_*P*_; partially immune, *S*_*S*_; and fully immune, *R*; vaccinated, *V*; primary infected, *I*_*P*_; secondary infected, *I*_*S*_. *α* and *ε* represent varying degrees of transmission of secondary infections and susceptibility of *S*_*s*_ relative to primary infections. *ν* is a constant fraction representing the fully and partially susceptible populations are vaccinated each week (thus entering the vaccinated class *V*). 1/*δ*_*vax*_ represent vaccinal immunity duration. Full immunity from natural infection is assumed to wane at rate *δ*, while immunity from vaccine wane at rate *δ*_*vax*_. The natural birth rate μ is assumed equal to the natural death rate. *s*_*vax*_ is a binary variable representing vaccination is introduced or not.

By assuming different values of immunity of secondary infections to SARS-CoV-2, *ε*, natural immunity duration, 1/*δ*, vaccinal immunity duration, 1/*δ*_*vax*_, and so on, this model simulated likely complex COVID-19 dynamics in the future and found that even an imperfect vaccine could contributed to control COVID-19 transmission, reduce subsequent peaks of clinically severe cases, and, if the number of susceptible individuals vaccinated reached a threshold, the outbreaks could be suppressed within 5 years. However, they also suggest that, a sustained immunization strategy is necessary to avoid greater outbreaks in the future.

At present, the beneficial effect of vaccine on COVID-19 transmission have been well recognized. However, vaccine for SARS-CoV-2 was still in severe shortage in many developing countries, raising the question that how to prioritize and allot available vaccine [15]. Bubar et al. developed an age-stratified SEIR model accounting for age structures in different countries to evaluate and generate vaccine prioritization strategies for SARS-CoV-2 [132]. After comparing five age-stratified prioritization strategies prioritized vaccines to different age groups, the study reported that prioritizing highly effective vaccine to individuals ages 20–49 years could minimize cumulative incidence, while prioritizing vaccine to individuals older than 60 years will have better effect on reducing mortality and years of life lost. Besides, if a vaccine has poor efficacy in older adults, vaccine prioritized to younger age groups would be more effective. Accounting for the seroprevalence and individual serological testing, further priority given to seronegative individuals could make better use of the available doses.

#### Identifying the start time of epidemic

By inserting the different possibilities of start time of the epidemic in the model, transmission dynamics model could be used to make estimation on the potential start time of an outbreak. On June 11, 2020, a local case was confirmed after 56 days of zero daily new cases in Beijing. Later, on June 15, there were totally 106 cumulated new cases. According to the case report, the earliest onset date of cases was June 4, indicating that the transmission had started even earlier. To explore the possible start time of this resurgent epidemic and estimate the effect of interventions, Wei et al. [69] developed an SEIR model assuming different start time of the epidemic to fit daily onset infections before June 12, when the interventions had not been introduced. They inferred that the start time of the re-emerged COVID-19 epidemic in Beijing was between May 22 and May 28, with the highest probability on May 25 (23%).

Similarly, Peirlinck et al. [73] proposed a SEIIR model, with an addition compartment of asymptomatic cases, and explored the most probable origin date of epidemic in Santa Clara County, the US, by the Nelder–Mead optimization method. They found that the initial outbreak date of epidemic in Santa Clara County could be traced back to January 20, 2020.

In addition to the above applications, transmission dynamics models have also contributed to other aspects in COVID-19 studies, such as estimating the treatment effects of antiviral drugs, exploring the possibility of reinfection, predicting the imported or exported cases of a specific country, exploring the possibility of COVID-19 outbreak in one country or region if there were imported cases [133], [134], [135]. For epidemics due to imported cases, similarly, transmission dynamics model could be used to explore how many initial infections transmitted SARS-CoV-2, which would help to find chains of transmission.

## Limitaions of transmission dynamics models

### Insufficient data limited the accuracy of model

**Figure 11:**
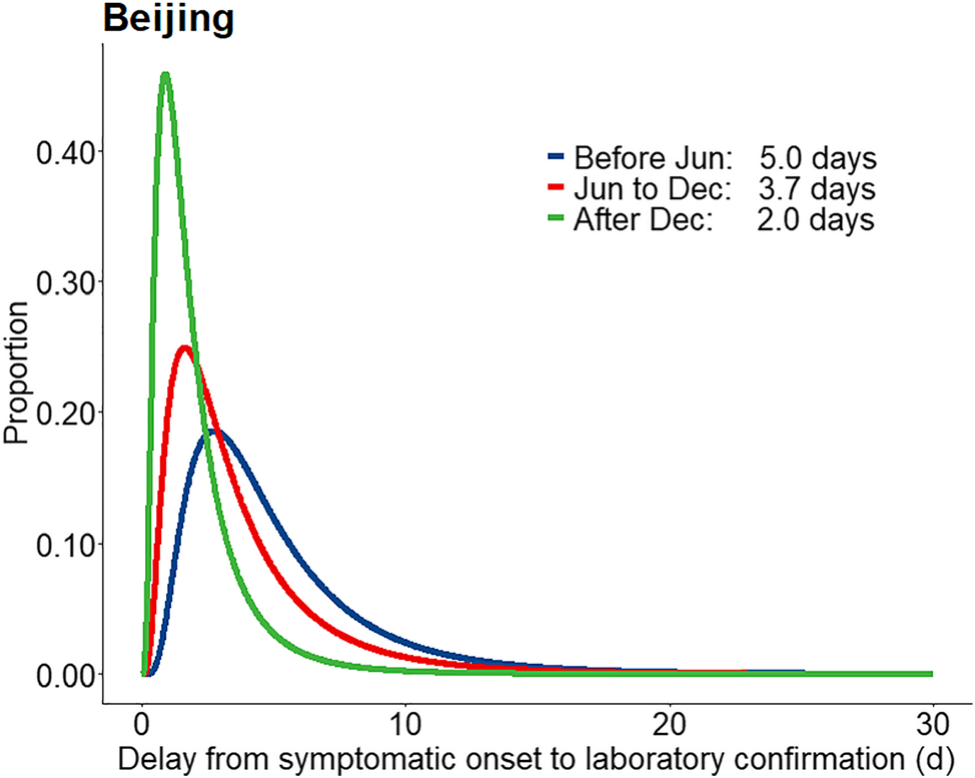
Delay from symptomatic onset to laboratory confirmation in Beijing.

It is a huge challenge to forecast future trends of emerging infectious diseases timely, accurately and reliably, especially at the early stage of the epidemic. In an ongoing epidemic, often there will be insufficient data for model construction and validation [136]. In studies on the COVID-19 epidemic, the most common used index may be the number of confirmed cases. However, at the early epidemic, nucleic acid testing capacity was insufficient, resulting in appreciable quantity of undetected cases and a delay from being infected to be confirmed. Even in Beijing, the capital of China, there was also a 2-day delay from infection to be confirmed in early 2021 (Figure 11). Thus, it is possible that models fitting the pandemic using data of confirm cases may generate biased estimation of crucial epidemiological parameters, and should be interpreted with caution. When fitting a transmission dynamics model, a reliable type of data is the number of onset cases developing symptoms. Besides, considering the inflow and outflow population of a city during an epidemic, mobility data is also needed.

### Limited knowledge of COVID-19 resulting in wrong structures of model

The accuracy of a transmission dynamic model is related to the knowledge of the SARS-CoV-2 virus itself. At the beginning of COVID-19 pandemic, scientists had limited information about the biologic features of transmission. For example, some early models ignored the incubation period which is defined as the interval from being infected to developing symptoms. Meanwhile, some studies inserted important parameters (e.g. the length of incubation period) in their models by borrowing information from SARS and MERS epidemics, which took the risk of ignoring the differences among the three kinds of virus. Nearly all early models ignored the reinfection of COVID-19, while recent studies revealed the possibility [137]. Furthermore, the infectiousness of asymptomatic cases and infected individuals in incubation period was ignored in early studies, which would result in biased results. Although early models are imperfect, they play an important role in understanding the characteristics and dynamics of COVID-19 at the beginning of epidemic.

### No models are completely accurate

Most of the transmission dynamics model for COVID-19 did an excellent job in reproducing past epidemic evolution but not in predicting future epidemic [138]. A review published in 2020 evaluated the deviations of predictions of published COVID-19 studies. They found that of the seven reviewed studies which made totally eight short-term predictions of infections which predict the future number of infections within 15 days, and only five of eight predictions were within the ±50% range around the true values. For 23 long-term predictions in 19 reviewed studies, only five of 23 predictions were higher the ±100% range around the true values [139]. Besides, this review summarized eight studies which predicted the peak time of regions in China and 40% of these peak time predictions had deviation days greater than or equal to 5 days compared with the actual peak time.

With increasing knowledge of SARS-CoV-2, more sophisticated models with extending compartments and parameters would be developed to better picture the dynamics of COVID-19 epidemic. However, given the same dataset of cases, more sophisticated models may not be more reliable and accurate, especially in prediction [140]. Simple models are easy to estimate the parameters but are possibly naive and unrealistic, while complex models may be more realistic but may contain parameters which are hard to estimate and obtain and may be overfitting [136, 140]. An overfit model cannot be generalized beyond the observed sample data. Setting fixed values of parameters, such as incubation period and protective effects of vaccine, based on virus studies and epidemiological studies seems a good way to reduce parameters need to be estimated. When formulating a transmission dynamics model for COVID-19, it is necessary to decide which compartments and parameters to include and which to omit depending on the questions to be answered.

In the current studies, model diagnostics for transmission dynamics models are largely lacking. As Johnson and Omland state, “Parsimony is, in statistics, a trade-off between bias and variance” [141]. A good model is a proper balance between underfitting and overfitting. When considering a collection of models, Akaike Information Criterion (AIC) would be a reliable model selection method, which accounting for the goodness of the fit and the principle of parsimony [142].

### The effect of exact intervention is hard to estimate but can in theory

It’s difficult for transmission dynamics models to estimate the effect of exact single intervention on COVID-19 transmission. The effects of control measures are often estimated by comparing values of transmission parameters, such as transmission rate, *R*_*e*_ and *R*_*t*_, etc., before introducing interventions to that after introducing interventions or comparing the observed numbers (case, hospitalization and death, etc.) to the expected number without the interventions. Nevertheless, the spacing time between implementing several interventions is so close that it’s difficult to estimate the individual effects of exact interventions [143]. Besides, the effects of control measures estimated by transmission dynamics models often contain the effect of human behavior change. As an example, when there is a lot of infection in the society people would be more careful and take self-protection actions dampening the transmission of COVID-19. Estimating this effect is extremely difficult. Thus, adding more parameters in modelling can improve the accuracy of model fitting theoretically, although it may not be possible for some factors and parameters to be realized in the early stage of an epidemic.

## Understanding the values of transmission dynamics model

Indeed, as a result of complex, rapidly changing and intensive interventions, quickly evolving virus, increasing understanding of SARS-CoV-2 and capability of diagnosis and treatment, predicting the future trend of the epidemic precisely, especially in a long future, is nearly impossible [144]. As an example, unless there is travel restrictions in some region, no one can accurately predict how the size of the total population will change in the future due to inflow and outflow population, leading to the uncertainty when we want to predict whether an outbreak of COVID-19 would occur if an infected individual entered the susceptible population. In addition, the extent of protective immunity and how long the recovered individuals would be protected remain uncertain. Failure to ascertain of how many individuals have been infected with no or mild symptoms also limits our ability to predict the future of COVID-19 pandemic.

Even predictions for the next 15 days are possibly not totally accurate due to rapidly changing situation, especially in the early pandemic. Nearly all the predictions are fitting the theoretical trend in the future under current settings of parameters. But given the considerable uncertainty due to underlying disease, climate and behavior change, not to mention the uncertainty over exactly what and when interventions would be implemented, the trajectories of the epidemic were quite different [145]. For example, on February 5, 2020, the National Health Commission of the People’s Republic of China announced the release of the tentative fifth revised edition of the Diagnosis and Treatment Plan for COVID-19 which resulted in a dramatically increase of the number of daily new cases of COVID-19 in China. This huge increase of confirmed cases as a result of change in diagnostic criteria is impossible to be forecasted by any models whatever data they used and whatever structure they had. Although more complex structure and parameters could help models reduce the bias of model fitting, we should be aware of the risk of overfitting. Meanwhile, in the early stage of epidemic, with limited knowledge of virus and cumulated data, it is almost impossible to have a stable solution. Some models may even fail to converge if too many parameters are specified in the model.

Given the same dataset of cases, a complex model may not be more reliable. At the beginning of a pandemic, the knowledge of disease was limited and a simple model would be more reliable. With increasing knowledge of virus and data, the simple model could be expanded with extending compartments and parameters and answered. Which parameter and compartment to be added is depending on the questions. For example, when studying the effect of control measures, a parameter representing the reduction on transmission rate or contact rate is needed. Transmission dynamics models with different compartments and parameters may lead to similar outcome in short-term predictions but not to do so in long-term predictions. For future prediction, it is appropriate to only make short-term predictions, fewer than 15 days in the future, as a result of fast-changing situations, such as the implementing of interventions and so on, and report predictive intervals for the model. Besides, accounting for the various situation, models with time-varying parameters such as transmission rate would be more reliable.

Understanding what models cannot predict is sometimes more important than understanding what they can [35, 145]. We summarized the values of transmission dynamics models into three categories:

– **Early warning of the epidemic.** By using the accumulated data during an outbreak, transmission dynamic models could be established. Even at the early stage of an outbreak when almost nothing of the novel pathogen was known, the models could help to explore possibility of epidemiological features and estimate related parameters with a reasonable range to facilite virologic studies and provides references for following studies [146]. With the increasing knowledge of SARS-CoV-2, mathematical models could be modified to fit the data and then predict the future trend of epidemic, such as the peak time and size, hospitalization needs, and so on. Although all these predictions could not be completely accurate, these predictions could give early warnings that how large the number of cumulated cases would be in the future under current condition when no more interventions were implemented. For example, at the beginning of epidemic, Li’s model had already estimate that the infectiousness of infected individuals with no or mild symptom is 55% infectiousness of cases with severe or critical symptom [13]. With the estimated transmission parameters including transmission rate, incubation period, etc., scientists can make early warning about the epidemic. At the end of the pandemic, the model could be used to explore the probability of resurgence if a series of interventions were lifted.
– **Decision Supporting.** Making decisions are always difficult, especially in an epidemic in which thousands even millions of people may be affected by the decisions. Strict control measures may save lives, but destroy economics and social stability. With the fitted transmission models, scientists can identify the influences of different parameters on transmission. We can then specify different values of the parameters so as to emulate how the disease will spread and how the epidemic will develop under different scenarios of control measures. It is also possible for us to obtain the optimal control strategies which balance the burden of economy and health system and controlling COVID-19 transmission. Thus, the models would be helpful when making “evidence-based” decisions.
– **Policy Evaluation.** After the implementation of comprehensive prevention and control measures, compartment models could also assist to quantitatively assess the effectiveness of these control measures by comparing the expected number and observed number of cases or the reduction on transmission rate so that the control measures could be adaptively adjusted in time when necessary, as well as explore appropriate control strategies. After the epidemic is over, the cost-effectiveness of prevention and control measures can be evaluated to provide evidence for government to establish an epidemic emergent response system [147].

To summary, we proposed that the core values of transmission dynamics models are not only to predict, but also to explore various possibilities of the epidemic.

All models are wrong, but some are useful.

– George E. P. Box, statistician
